# Measuring the social support network in autistic clients: development and validation of the Network in Action-Interview

**DOI:** 10.3389/fpsyg.2025.1411908

**Published:** 2025-04-03

**Authors:** Rinske M. van den Heuvel, Hilde M. Geurts, Michel Wensing, Jan-Pieter Teunisse

**Affiliations:** ^1^Leo Kannerhuis, Youz (Parnassia Group), Oosterbeek, Netherlands; ^2^Lectoraat Volwaardig Leven met Autisme, HAN University of Applied Sciences, Nijmegen, Netherlands; ^3^Department of Psychology, University of Amsterdam, Amsterdam, Netherlands; ^4^General Practice and Health Services Research, University Hospital Heidelberg, Heidelberg, Germany

**Keywords:** autism, adults, mental healthcare, social network, social support

## Abstract

**Introduction:**

As social relationships are intertwined with mental health recovery, it is important to address a client’s social support network during mental health interventions. This seems even more important for autistic clients, because research suggests they have on average smaller networks and experience more loneliness than non-autistic individuals. Therefore, an interview assessing the social support network in relation to intervention goals was co-created together with stakeholders (autistic clients, mental healthcare professionals and a mother of an autistic client). In addition, the psychometric properties and acceptability of this Network-in-Action-Interview (NiA-I) were studied as pre-registered (AsPredicted #59767).

**Methods:**

The Nominal Group Technique was used to co-create the NiA-I with stakeholders and it was administered to autistic clients (*n* = 44) recruited in a highly specialized mental health facility.

**Results:**

Network-in-Action-Interview social support scores were significantly correlated with the Multidimensional Scale of Perceived Social Support, indicating sufficient convergent validity. Clients and professionals reported that the NiA-I provided the therapist with greater insight into the client’s social support network. Professionals reported the NiA-I could be improved regarding administration duration.

**Discussion:**

This cross-sectional study shows that the NiA-I is a solid and helpful tool for including the social network in clinical practice. Addressing and including a client’s social support network is important for recovery-focused mental health treatment. The NiA-I can assist professionals in taking such actions.

## 1 Introduction

A person’s social support network can either promote or hinder recovery from mental health problems (Reupert et al., 2015). For example, on the one hand having meaningful social connections is one of the core aspects of personal recovery (Tew et al., 2012), while on the other hand distancing oneself from certain others who are disempowering or overprotective can also promote recovery (Reupert et al., 2015). Therefore, it is important to pay attention to a client’s social support network during mental health interventions and how this support relates to the individual’s intervention goals. In this study, we focus on developing and studying a co-created interview that assesses social support aspects in relation to intervention goals.

Regarding social support, autistic individuals appear more vulnerable. For example, autistic adults experience more loneliness (Moseley et al., 2021; Tobin et al., 2014) and lower levels of social support (e.g., Moseley et al., 2021) compared to non-autistic individuals, with the latter being associated with lower quality of life (Charlton et al., 2022). Moreover, wishes related to their social networks were reported by the majority of autistic adults recruited via a mental health facility (van den Heuvel et al., 2022). This strengthens the need of considering the social support network during mental health interventions in autistic individuals, a group known to have an elevated prevalence of mental health problems such as depression and anxiety disorders (Hollocks et al., 2019; Hudson et al., 2019).

However, social network interventions are not a one-size-fits-all solution, as everyone has different needs which differ along the journey of recovery (Tew et al., 2012). Using a social network mapping tool can assist the mental healthcare professional and the autistic individual in discovering collaboratively what could help the client regarding their network in several ways. For example, social network mapping can help to gain a broader understanding of the client’s daily life including their social interactions (Brooks et al., 2022). Also, it opens a conversation to empower the client (Sweet et al., 2018) which suits within a contextual-focused, recovery-oriented mental healthcare. In addition, such a tool facilitates the identification of strengths and resources within one’s social environment (Tew et al., 2012), which might serve as a first step in assisting clients to mobilize support within their network. Lastly, it can help in identifying suitable network members for involvement during mental health interventions, which is associated with better client-reported intervention outcomes and higher satisfaction with care in clients with psychiatric conditions (Svendsen et al., 2021).

Based on these arguments plus a lack of suitable existing instruments for clinical practice, we developed the Network in Action-Questionnaire (NiA-Q) (van den Heuvel et al., 2022). This is a digital questionnaire assessing both functional (e.g., perceived social support and interpersonal distress) and structural aspects (e.g., network size) of the social support network. These aspects are assessed in relation to the individual’s treatment goals, so the information from the NiA-Q is directly applicable to the individual’s mental health intervention. Therefore, it is expected this instrument will be more suitable for use in clinical practice compared to other instruments that lead to a more general impression of someone’s social network. However, using the NiA-Q in clinical practice revealed that both clients and professionals encountered some challenges. That is, some clients considered their social network a too sensitive topic for an anonymous, digital questionnaire without the opportunity to discuss this directly with their practitioner. In addition, the professionals missed opportunities to respond to the client’s needs, for example, by explaining if something was unclear or by asking further questions on specific topics to gather as much relevant information as possible.

Therefore, in this study, we transformed the NiA-Q into an interview version, the Network in Action-Interview (NiA-I), in collaboration with stakeholders using a structural group technique. In addition, this study investigated (1) the first psychometric properties of the newly developed NiA-I; and (2) the acceptability of the instrument to autistic clients and professionals.

## 2 Methods

### 2.1 Community involvement

Throughout the study, a project group was involved consisting of two autistic clients, a mother of an autistic client and five healthcare professionals from diverse backgrounds working in an autism mental healthcare center. They provided input during the development of the NiA-I, on recruitment strategies, and on content of the acceptability measures.

### 2.2 Development of network in action interview

The development process of the NiA-I consisted of three steps. In the first step, the NiA-Q (van den Heuvel et al., 2022), was transformed into a first draft version by the researchers. A limited number of questions that were considered relevant based on literature on social network concepts were added. For example, a question about the relationships between network members was added to the interview to obtain a measure of network density. Next, this version was presented to the project group using the Nominal Group Technique (NGT; Delbecq and Van de Ven, 1971; McMillan et al., 2014) during an online meeting (duration: 120 min). NGT facilitates the generation of ideas about a specified topic, by structuring group discussion in four phases to reach a collaborative decision (McMillan et al., 2016). NGT minimizes possible adverse effects of intragroup dynamics and encourages openness and contributions from all group members (Hatch et al., 2021). The researchers participated as facilitators. Following NGT procedure, during the Silent Generation phase, all members silently wrote down their suggestions for modifications to the proposed interview. Subsequently, during the Round Robin phase, members named their suggestions. In the Clarification phase, members could discuss and group double or overlapping suggestions. The last phase, Ranking, was conducted by e-mail: all members voted for each suggestion whether they considered this a useful suggestion or not. Votes from the autistic individuals and the mother of an autistic client counted double to create more balance in the votes of clinical professionals versus experts by experience. Suggestions that more than half of the group voted as being beneficial were implemented by the researchers in a new version of the NiA-I.

In the second step, the NiA-I as it emerged from the NGT meeting was presented to other professionals (*n* = 10) and autistic clients (*n* = 4) in individual feedback sessions with a researcher (duration: 45 min). Participants were asked how item wording could be improved and what topics were missing.

In the third step, feedback gathered in the second step was presented to the project group in a second NGT session (duration: 120 min). Therefore, the Silent Generation and Round Robin phases were not applicable. Clarification and Ranking phases proceeded in the same manner as the first NGT session, resulting in a version of the NiA-I that was pilot tested by four autistic clients and their therapists. Based on their experiences, final adjustments were made to the NiA-I in consultation with the project group. Examples of adjustments were changes in the order of questions and improvements in layout. Also, the suggestion for therapists to look up some information (i.e., on treatment goals and current care providers) before interview administration was added, as this decreased the administration time and load to clients. See Table 1 of the topics covered in the final version of the NiA-I. As part of the interview, participants make a sociogram of their current and desired situation regarding their social network. The network members are placed in one of four concentric circles around the client, with the innermost circle representing the most supportive or significant individuals. This structure helps to visualize the perceived closeness or importance of each network member and desired changes.

**TABLE 1:** Description of areas covered in the Network in Action-Interview.

Item	Item description
1	List of important persons or groups of persons and their interrelationships (i.e., alter-alter relationship)
2	Current situation of how supportive or important network members of item 1 are, represented by placing in one of four circles around client (i.e., sociogram)
**3**	**Indication of level of practical, informational, emotional and general social support per treatment goal, provided by whom plus whether current support is sufficient. Also: level of perceived hindrance from the network per treatment goal, related to who and in what way**
4	Type of relationship with each network member and who usually initiates contact
5	Network with professionals and satisfaction rating with services
6	Persons or groups to whom client provides support and by what means
7	Clients’ preferences about network involvement in (treatment) goals, and if desired: in what manner
8	Satisfaction rating with current network
9	Desired situation of social network using circles of item 2, followed by questions on what the client needs to achieve the desired changes
	**Concluding question: What did you think was the most important thing we discussed in this interview?**

Items in bold are analyzed in this manuscript.

### 2.3 Participants

All autistic clients and their therapists were recruited via a highly specialized autism mental health facility in Netherlands. The client population is characterized by having co-occurring conditions besides a clinical diagnosis of an autism spectrum disorder (ASD) and they have often had previous treatment that was unsuccessful. Data collection took place in 2021 and 2022. Inclusion criteria for the autistic individuals were (1) ASD diagnosis based on DSM-5 (American Psychiatric Association, 2013); (2) being a client of the specialized autism center; and 3) aged 14 years or older. There were no specific inclusion criteria for therapists.

### 2.4 Procedure

The study was classified as not falling under the Dutch Act on Medical Research Involving Human Subjects (WMO) (reference number W20_358 # 20.397) by the Medical Ethics Committee of Amsterdam University Medical Center, and was approved by the Ethics Committee of the Psychology Department of the University of Amsterdam (2020-EXT-12318; 2021-EXT-13059). Recruitment took place through multiple strategies: clients were asked to participate either (1) by their therapist during treatment, who approached clients they deemed eligible; (2) by a therapist after the intake procedure; (3) by the researcher in a therapeutic group meeting. The NiA-I was administered by either a therapist of the client as part of ongoing treatment (*n* = 19) or the researcher (*n* = 25). The interview was conducted face-to-face (*n* = 41), online (*n* = 2) or mixed (*n* = 1) in one or two sessions. The administration of the NiA-I lasted between 30 and 120 min (M = 69.9; SD = 25.6; median = 60), with 5–30 min (M = 17.2; SD = 9.5; median = 15) of preparation time for the professional. The client and therapist completed the acceptability measure directly after NiA-I administration. The client completed the Multidimensional Scale of Perceived Social Support (MSPSS), Autism Quotient (AQ) and Social-Responsiveness Scale (SRS) via a secured online platform within the following week.

### 2.5 Measures

#### 2.5.1 NiA-I

The NiA-I consisted of nine main questions on various aspects of the social network, which were divided into sub-questions (see Table 1). Of these, question 3 assesses perceived social support through a quantitative score. The scores of the sub-questions of question 3 were averaged to determine a NiA-I social support score that is used for analyses of convergent validity and internal consistency. In this question, four aspects of social support were asked for a maximum of three separate treatment goals. If clients had fewer than three current goals, the four items were answered only for these goals. Answers were scored on a five-point scale (1 = not at all; 5 = very much) or as “not applicable.” The NiA-I is freely available upon request from the first author (NB the instrument is in Dutch). No additional training is needed prior to administration by a clinical professional.

#### 2.5.2 Multidimensional Scale of Perceived Social Support (MSPSS)

The MSPSS (Zimet et al., 1988) measures the perceived level of social support from friends, family and significant other/special person. It consists of 12 items measured on a seven-point scale. Higher scores indicate a higher level of social support. The psychometric properties of the MSPSS have been demonstrated in a range of studies, including its reliability and validity of the Dutch version (Pedersen et al., 2009) and in both adolescent and adult samples (e.g., Bruwer et al., 2008; Zimet et al., 1990).

#### 2.5.3 Acceptability ratings

To measure client’s and therapist’s experience with the NiA-I, we developed acceptability ratings with the project group. The five-item client-version and the six-item therapist-version largely overlapped in content (see Table 3) and both versions were measured on a five-point scale (1 = very strongly disagree; 5 = very strongly agree).

#### 2.5.4 Social-Responsiveness Scale-2 (SRS-2)/Social-Responsiveness Scale-A (SRS-A)

Depending on the participant’s age, the caregiver-report SRS-2 (Constantino and Gruber, 2005) or its adult version, the self-report SRS-A (Constantino, 2002), was used. It measures autistic traits in 64 (SRS-A) or 65 (SRS-2) items, which are answered on a four-point Likert scale and lead to scores between 0 and 3. The total scores range from 0 to 192 for SRS-A and from 0 to 195 for SRS-2. Higher scores indicate more autism traits. We used the cut-off score of 57 for the SRS-2 and 54 for the SRS-A. Mean scores for non-autistic people have shown to range between 37.7 and 76.5 (Bezemer et al., 2020). The validity and reliability of the Dutch version of the SRS-2 and SRS-A have shown to be adequate (Noens et al., 2012; Roeyers et al., 2011). The SRS-2/SRS-A score was used for descriptive purposes and for subgroup analyses, see section “2.6 Data analysis.”

#### 2.5.5 Autism Quotient (AQ)

The AQ (Baron-Cohen et al., 2001; Hoekstra et al., 2008) measures characteristics indicative for autism with a cut-off score of 32. It consists of 50 items answered on a four-point Likert scale. A higher score indicates more autism characteristics. Answers are dichotomized into 0 (not indicative of ASD) or 1 (indicative of ASD) (Baron-Cohen et al., 2001), leading to a possible score range of 0–50. Mean scores for non-autistic people have shown to range between 16.4 and 22.2 in the general population and between 20.9 and 33.1 in psychiatric samples (Bezemer et al., 2020). The Dutch version of the AQ is reliable and valid (Hoekstra et al., 2008). The AQ score was used for descriptive purposes and for subgroup analyses, see section “2.6 Data analysis.”

#### 2.5.6 Adherence

Only in three of 19 NiA-I takings by therapists (16%) audio recording succeeded. Therefore, the recordings were not formally evaluated. No problems with administration occurred in these three recordings. Additionally, no problems were reported by clients or therapists during other, non-recorded administrations, nor did clients express objections to specific items.

### 2.6 Data analysis

The study design and analyses were pre-registered on AsPredicted (#59767^1^). To investigate convergent validity, Pearson correlation between the NiA-I question 2 mean score and MSPSS mean score was calculated. Both a frequentist approach (i.e., leading to a *p*-value) in R Core Team (2017) and Bayesian statistics in JASP version 0.13.1 (JASP Team, 2020) were applied. For the latter, the prior was kept at the default (i.e., 0.707). We report the Bayes Factor_10_ (BF_10_), which indicates the probability that the alternative hypothesis is true compared to the null hypothesis. The correlation analysis was repeated including only participants scoring above either the AQ cut-off (i.e., 32) or SRS-2 (i.e., 57)/SRS-A cut-off (i.e., 54) to check whether this changed the results. In addition to the correlation analysis, a Bland–Altman plot was used to inspect systematic differences across the range of scores (Bland and Altman, 1986), such as whether differences in scores tend to occur at lower or higher score ranges. First, the NiA-I and MSPSS scores were transformed into z-scores to enable cross-measure comparison. Second, the difference value of each NiA-I-MSPSS pair was plotted on the y-axis and the mean value for each NiA-I-MSPSS rating pair was plotted on the x-axis. A positive mean difference score indicates a pair where the NiA-I score is higher than the MSPSS value.

Internal consistency for the social support scores of the NiA-I was presented in Cronbach’s alpha. Since some participants had less than three treatment goals and not applicable was a valid answer option if a social support aspect did not apply to a certain intervention goal, we followed the procedure described by Arifin (2018) that takes not applicable answer options into account.

Acceptability ratings of clients and professionals were presented in descriptive statistics. As professionals could administer the NiA-I for multiple clients and therefore complete the acceptability measure multiple times, we calculated intraclass correlations (ICC) for the ratings of professionals.

Lastly, we briefly describe the responses on the final, open-ended question of the NiA-I for explorative purposes.

## 3 Results

### 3.1 Sample characteristics

As pre-registered, 44 clients participated in the study. Clients were between 14 and 58 years old (*M* = 23.8, *SD* = 8.8). Regarding gender, 17 (38.6%) clients identified as female, 25 (56.8%) as male and two (4.5%) as other. See for further characteristics Table 2.

**TABLE 2:** Sample characteristics.

Characteristic	Total sample (*N* = 44)
Treatment setting *n* (%)	
Outpatient	20 (45.5)
Day treatment	19 (43.2)
Inpatient	5 (11.4)
Treatment duration in weeks *M* (*SD;* range)	23.4 (17.3; 0–68)
AQ total score *M* (*SD;* range)	26.5 (8.1; 10–41)
SRS-2 total score *M* (*SD;* range)	110.0 (29.0; 60–138)
SRS-A total score *M* (*SD;* range)	113.8 (22.0; 72–156)

AQ, Autism Quotient; SRS-2, Social-Responsiveness Scale-2; SRS-A, Social-Responsiveness Scale-Adult.

### 3.2 Psychometric properties

#### 3.2.1 Convergent validity

**FIGURE 1:**
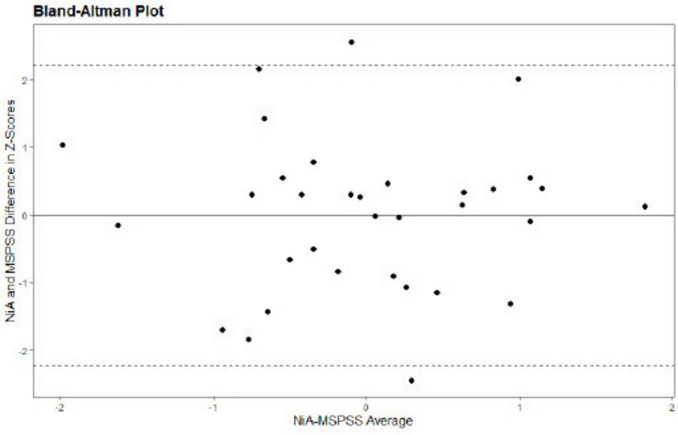
Bland-Altman plot shows no systematic differences between Network in Action-Interview and Multidimensional Scale of Perceived Social Support Scale.

Social support items on the NiA-I and MSPSS were moderately positively correlated, *r*(31) = 0.36, *p* = 0.042, BF_10_ = 1.57. The Bayes factor indicates anecdotal evidence that the two scores are correlated (Wagenmakers et al., 2011). Conform pre-registration, we planned to repeat the correlation analysis with solely those participants scoring above cut-off score on either AQ or SRS-2/SRS-A. However, no participants had to be excluded based on this restriction. The Bland-Altman plot showed no systematic differences across the range of social support scores of the NiA-I and MSPSS (see Figure 1).

#### 3.2.2 Internal consistency

The social support items of the NiA-I had a good internal consistency (Evers et al., 2009), α = 0.85.

### 3.3 Acceptability ratings

Acceptability ratings of clients and professionals are presented in Table 3. As three professionals participated two or three times, ICCs were calculated (see Table 3). For three items (i.e., duration, clarity of questions, and compatibility with clinical practice) ICCs showed that all variance could be attributed to variance between different raters, so the professionals who participated more than once were consistent in their scoring across clients. For the other items, ratings of professionals varied when they administered the NiA-I to multiple clients.

**TABLE 3:** Acceptability ratings for Network in Action-Interview (NiA-I) items.

Item description	Client (C)				Professional (P)				
	***M***	***SD***	**% Agree or very strongly agree**	***n* ^1^**	***M***	***SD***	**% Agree or very strongly agree**	***n* ^2^**	**ICC**
1. The duration is acceptable.	4.02	1.03	74.4	43	3.41	1.23	58.8	17	1
2. The questions are clear.	4.16	0.69	88.4	43	3.88	0.86	82.3	17	1
3. The information resulting from the interview is useful for treatment.	3.76	0.96	66.7	42	3.92	0.80	73.0	26	0.61
4. The interview gives my therapist/me as a therapist more insight into the social network.	4.35	0.79	82.4	17	4.27	0.83	84.6	26	0.32
5. C*:* gives me more information about my social network. / P*:* is easy to work with.	3.88	1.12	62.8	43	4.12	0.78	76.5	17	0.61
6. P*:* fits well within clinical practice.	N/A	N/A	N/A	N/A	3.56	0.89	52.9	16	1
All items combined.	4.00	0.60	N/A	43	3.95	0.66	N/A	26	0.36

^1^If the researcher administered the Network in Action-Interview (NiA-I), item 4 was considered not applicable for clients to fill out.

^2^If the researcher administered the NiA-I, only item 3 and 5 were filled out by the professional. NB, The range of all acceptability items was 1 (*very strongly disagree*) to 5 (*very strongly agree*).

### 3.4 Exploratory analyses

In order to obtain qualitative information, we examined the open-ended, final question of the NiA that asked clients what they thought was the most important topic discussed during the interview. Around a quarter of participants (*n* = 12) described their desired changes regarding their social network as the most important topic. Some clients (*n* = 6, 14%) explicitly referred to the network circles of question 2 and 9 and how it helped to formulate their wishes:

*“Placing people in the circle (from step 2). I realized that I feel I have to approach everyone in the same way and meet all their expectations, but this may not be as necessary with people who are a bit more distant from me.” (23 years-old woman)*

Other participants (*n* = 9, 20%) mentioned that answering concrete questions about their network provided them with insight:

*“It’s kind of unusual to look at my network in such a focused way. But it gives me a different angle and helps me think about it more broadly” (23 years-old man)*

Other examples of insights or topics that clients referred to in their answers to this question are:

*“The question about who you want to involve; I would like my parents to listen to me more when I am struggling with something.” (21 years-old woman)*

*“The most important thing: family comes first. I also want to evaluate each of my contacts to see whether it drains or gives me energy.” (40 years-old man)*

*“I thought at first that I’d need to find people, but there are already enough people I can go to.” (21 years-old man)*

Eight clients (18%) did not answer this question.

## 4 Discussion

The purpose of this study was to co-create an interview based on the NiA-Q (van den Heuvel et al., 2022) with various stakeholders so that clinicians could use it to gain insight into an autistic client’s social support network. The results indicate that the NiA-I is a sufficiently valid, reliable, and acceptable instrument to assess the social support network in clinical practice in autistic individuals.

The results of acceptability ratings from clients and professionals showed that the NiA-I was considered relevant by the majority of both clients and therapists. Still, we have two suggestions to increase the usefulness of the NiA-I. First, it would be interesting to further explore the characteristics of clients for whom the NiA-I might be most beneficial, in order to better predict to whom and when this instrument is indicated. Second, the perceived usefulness for clients could be improved by adding a more comprehensive explanation of how the social support system can contribute to mental health recovery so that individuals could more easily relate the NiA-I outcomes to their own situation.

The NiA-I provided the therapist with more insight into the client’s social support network, which is an important goal of the instrument. However, the acceptability ratings of professionals also indicated that the NiA-I could be improved regarding administration duration and how the interview fits within current clinical practice. Therefore, shortening the NiA-I could be considered in future research, allowing the professional and/or client to choose between a full or shortened version based on the individual’s needs.

The convergent validity analysis indicates that the NiA-I and MSPSS (Zimet et al., 1988) social support scores are sufficiently associated but do also differ. A first explanation is that the MSPSS might focus more on emotional aspects of social support than on concrete or informational support, as previously noted by others (Tracy and Whittaker, 1990), whereas the NiA-I explicitly asks about both emotional, informational and practical support in relation to intervention goals. Although there were several reasons to choose for the MSPSS in this study (e.g., good psychometric properties, a validated Dutch version, suitable for use in both adolescents and adults), the difference in broadness of included social support features in the two measures could explain the moderate correlation.

However, another explanation is that social support related to intervention goals is considerably different from the construct of general social support, which is one of the reasons why the NiA-I was developed in the first place. For example, not all network members perceived as supportive in general are relevant for certain intervention goals. This leads to the more fundamental question of which aspects within the broad concept of social support contribute most to mental health recovery (Priebe et al., 2014), which might even be different for autistic individuals. Social interactions and subjective social support appeared related to psychological health aspects of quality of life in autistic adults, but not instrumental social support (Charlton et al., 2022). Future studies should explore such influences on mental health *recovery* in autistic adults, so that we can better understand which aspect of social support we can try to act on to improve outcomes for autistic individuals, a group with high prevalence of mental health problems (Hollocks et al., 2019; Hudson et al., 2019).

An advantage of the NiA-I is that it supports the strong motivation of clinicians^2^ to include the social network of an autistic client. Moreover, the tool offers a practical solution to working network- and recovery-oriented, for example, by incorporating it into a mental healthcare team’s working routine. This is relevant for practice, because research on family involvement in mental healthcare has pointed at the role of organizational policy and working routine for successful implication (Eassom et al., 2014; see text footnote 2).

Besides assisting professionals in using a network-oriented approach during mental health interventions, there are at least two other specific applications of the NiA-I. First, the information resulting from the NiA-I can provide a starting point for a personalized social support intervention. Social support interventions for persons with mental health problems are effective only if they have a personalized approach (Beckers et al., 2022), so if they take into account the needs of the individual client as is done with the NiA-I. The finding that social support interventions are effective is promising, because these have been suggested as an approach to increase wellbeing in autistic individuals (Bishop-Fitzpatrick et al., 2018; Moseley et al., 2021). As a second application, the NiA-I can help the client nominate members of a resource group (Nordén et al., 2012): a client-chosen group of key network members and professionals that meets regularly and supports the achievement of the client’s goals. Further research can explore what role the NiA-I can play in these two applications.

There are some limitations to keep in mind when interpreting the results of this study. First of all, this study included a relatively limited number of participants, so the psychometric properties of the NiA-I should also be evaluated in a larger sample. Future studies could assess other psychometric properties besides convergent validity and internal consistency. An interesting avenue for further research would be to explore predictive validity, for instance, by examining whether NiA-I scores are associated with recovery progress or perceived loneliness over time. A second limitation is that participants were not randomly selected, but were approached if their therapist expected a NiA-I to be relevant to them or if the client was interested in participating. This might have influenced the acceptability results. However, a nuance here is that a clinician in regular clinical practice will also first make a clinical judgment before administering the NiA-I to a client.

Strengths of the study are that different types of stakeholders were involved in the development of the interview. Additionally, we used a structured group approach in the development process using NGT (Delbecq and Van de Ven, 1971), which encouraged all participants of the project group to give their input in an equal manner.

To conclude, it is important to provide attention to social support and involve the social network during mental health interventions, especially in autistic clients given vulnerabilities in this area. The NiA-I provides a tool to practically implement this aim into the clinical working routine, resulting in greater understanding of wishes, needs and opportunities within the client’s social support network for both therapist and client.

## Data Availability

The datasets presented in this article are not readily available because they involve privacy-sensitive data that may be identifiable to individuals. Requests to access the datasets should be directed to RMvdH, r.vandenheuvel@leokannerhuis.nl.

## Footnotes

1
https://aspredicted.org/5CS_Q32

2 van den Heuvel, R. M., Wensing, M., Geurts, H. M., and Teunisse, J. P. (2023). *Network Involvement in Mental Healthcare for Autistic Adults – A Study in Healthcare Professionals*. Submitted.
